# Intravascular Ultrasound–Guided “Move the Cap” Technique for Heavily Calcified Ostial Chronic Total Occlusion

**DOI:** 10.1016/j.jaccas.2026.108145

**Published:** 2026-05-05

**Authors:** Kiwamu Sudo, Masakazu Yasuda, Kota Tanaka, Tomohiro Yamasaki, Yoshitomo Tsutsui, Yuta Nishimura, Satoshi Watanabe, Mutsumi Iwamoto, Heitaro Watanabe, Atsunori Okamura

**Affiliations:** Cardiovascular Center, Sakurabashi Watanabe Advanced Healthcare Hospital, Osaka, Japan

**Keywords:** antegrade dissection re-entry, chronic total occlusion, intravascular ultrasound–guided wiring using the tip-detection method, “move the cap” technique

## Abstract

**Background:**

The “move the cap” technique is used to overcome ambiguous or impenetrable proximal caps in chronic total occlusion (CTO).

**Case Summary:**

We describe a case where intravascular ultrasound (IVUS)–guided wiring enabled the successful treatment of a left circumflex ostial CTO with an impenetrable proximal cap.

**Discussion:**

An impenetrable proximal cap is an independent predictor of failure to cross with a guidewire during percutaneous coronary intervention for CTO. Our experience indicates that the “move the cap” technique can be performed more safely and effectively with IVUS-guided wiring.

**Take-Home Messages:**

An impenetrable proximal cap in CTO is linked to poorer outcomes in percutaneous coronary intervention. IVUS-guided wiring using the tip-detection method enables safer, more reliable application of the “move the cap” technique on proximal caps that are impenetrable.

## Visual Summary

**Figure:**
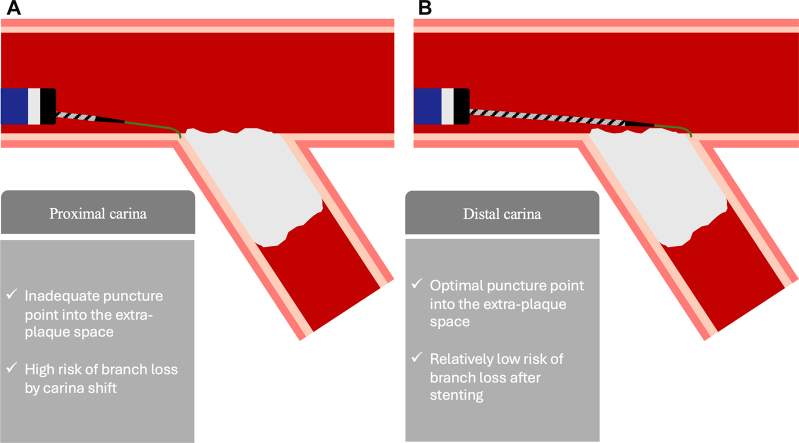

Despite advances in percutaneous coronary intervention (PCI) techniques, chronic total occlusion (CTO) PCI remains technically challenging with suboptimal success rates.^1^ Heavy calcification is a well-recognized predictor of adverse outcomes, including guidewire crossing failure, device delivery failure, and reduced long-term patency.

**Visual Summary:**
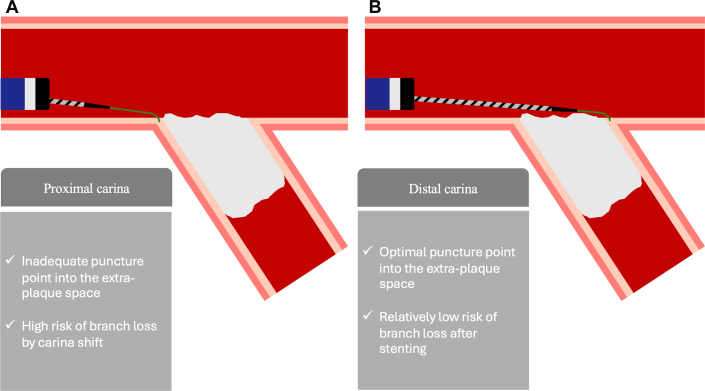
Adequate Puncture Point When the “Move the Cap” Technique Was Performed Around the Bifurcation (A) The proximal carina is not an ideal puncture site, as stenting may cause carina shift and increase the risk of occluding side branches. (B) The distal carina represents an optimal puncture site, minimizing the risk of side-branch compromise due to carina shift.

The “move the cap” technique is an effective strategy for ambiguous or impenetrable proximal caps. This method comprises 2 principal techniques. The first is the balloon-assisted subintimal entry technique, in which balloon dilation of the proximal CTO creates an extraplaque space for guidewire entry. The second is the scratch-and-go technique, where a high-penetration-efficacy guidewire is used to access the extraplaque space in the proximal CTO segment, followed by exchange to a polymer-jacketed guidewire to enable advancement while bypassing the proximal cap.^2^ A modified approach, the side balloon-assisted subintimal entry technique, creates a tear into the extraplaque space by balloon dilatation of a proximal side branch, thereby enhancing microcatheter stability during penetration.^3^ After guidewire entry into the extraplaque space, lesion crossing is attempted using antegrade dissection and re-entry (ADR) or retrograde dissection and re-entry.

A major limitation of the “move the cap” technique is limited control over entry into the extraplaque space, which may lead to expansion of the extraplaque space and compromise of side branches.

Intravascular ultrasound (IVUS)–guided wiring using the tip detection method (IVUS-based 3D wiring) has been widely adopted in CTO PCI, particularly in Japan, and can facilitate multiple procedural steps. Fine advancement and retraction of the IVUS motor drive unit mounted on the first guidewire, together with visualization of the shaft and tip of the second guidewire, can be visualized in real time, enabling precise targeting and puncture.^4^

We present a CTO case with an impenetrable proximal cap in which IVUS-based 3D wiring enabled the intentional application of the “move the cap” technique, followed by successful revascularization with tip detection–guided ADR (TD-ADR) using an antegrade-only strategy.

## History of Presentation

A 70-year-old man with 2-vessel coronary artery disease, including severe stenosis of the left anterior descending artery (LAD) and an ostial CTO of the left circumflex artery (LCX), underwent PCI on the LAD lesion 1 month earlier. However, his anginal chest pain persisted, and PCI was subsequently performed for the LCX CTO.

**Take-Home Messages**

• An impenetrable proximal cap in chronic total occlusion is associated with worse outcomes in percutaneous coronary intervention.
• Intravascular ultrasound–guided wiring with the tip detection method enables safer, more reliable use of the “move the cap” technique on proximal caps that are impenetrable.

## Past Medical History

The patient had undergone PCI for the LAD 12 years earlier and for the right coronary artery 10 years earlier. An additional PCI attempt for the LCX CTO had been performed 10 years earlier; however, antegrade wire crossing was unsuccessful because of severe calcification at the CTO entry site.

## Differential Diagnosis

The differential diagnoses included heart failure, pulmonary embolization, aortic dissection, and pneumothorax.

## Investigations

Electrocardiography revealed no significant St-T wave abnormalities. Transthoracic echocardiography demonstrated a left ventricular ejection fraction of 60% with mild hypokinesis of the posterior wall.

## Management

**Figure 1:**
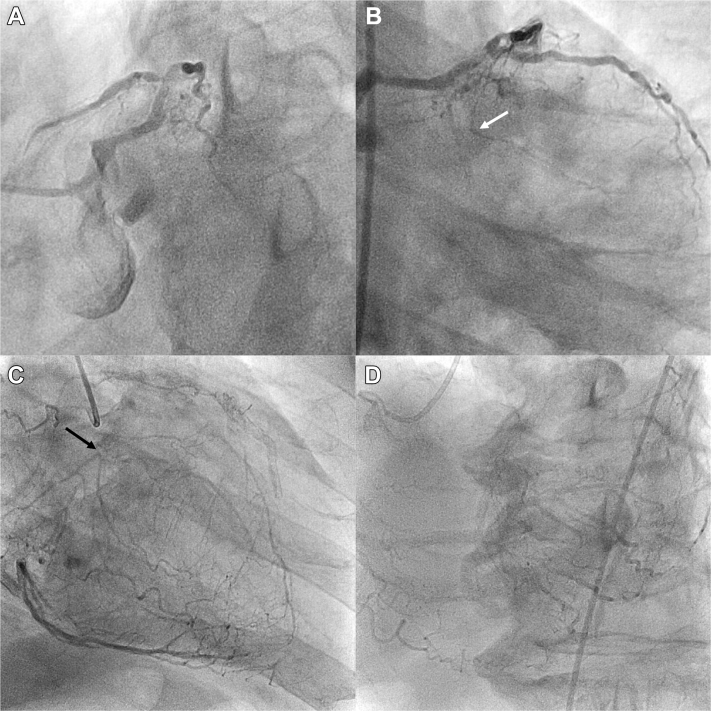
Baseline Coronary Angiography (A) The left circumflex artery was completely occluded at the ostium, with no stump. (B) Bridging collateral channels connected to the obtuse margin branch, the distal end of the first chronic total occlusion. The white arrow indicates the obtuse margin branch. (C) The distal end of the second chronic total occlusion was located at the bifurcation. The black arrow indicates the second chronic total occlusion distal end. (D) Tiny epicardial collateral channels from the right coronary artery to the left circumflex artery.

Preprocedural coronary angiography demonstrated a flush ostial occlusion of the LCX (Figures 1A and 1B, Videos 1 and 2) with a double CTO. The distal cap was located at the posterolateral branch bifurcation, with severe calcification throughout the CTO segment. The first CTO was short and was supplied distally by a bridging collateral channel (Figure 1B), whereas the second CTO extended from the obtuse marginal branch to the posterolateral branch (Figure 1C, Video 3). A retrograde approach was considered unsuitable because of the small caliber and tortuosity of the collateral channel from the right coronary artery; therefore, an antegrade approach was planned (Figure 1D, Video 4).

**Figure 2:**
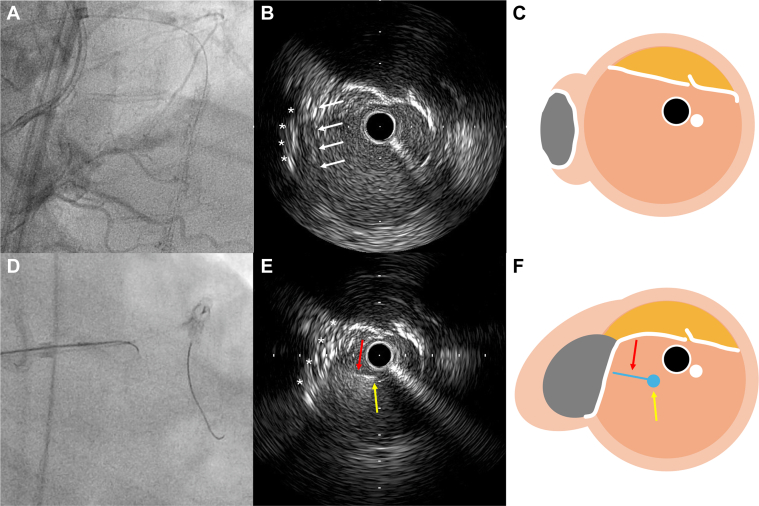
Coronary Angiography and Intravascular Ultrasound Image of the Procedure (A) Extremely tortuous and tiny epicardial collateral channels connected to the chronic total occlusion distal end. (B) Intravascular ultrasound image of the left circumflex artery chronic total occlusion entry from the left anterior descending artery. Massive calcification completely occupies the left circumflex artery chronic total occlusion entry site. The white arrow indicates the left circumflex artery chronic total occlusion entry; and asterisk, calcification. (C) Intravascular ultrasound annotations. The white line indicates superficial calcification; gray area, left circumflex artery chronic total occlusion; and yellow area, plaque behind calcification. (D) High-penetration-efficacy guidewires could not puncture the calcified entry under real-time intravascular ultrasound guidance because of their hardness. (E) Intravascular ultrasound image when a high-penetration-efficacy guidewire punctured the chronic total occlusion entry. The red arrow indicates the guidewire tip; yellow arrow, guidewire shaft; and asterisk, calcification. (F) Illustration of the guidewire position relative to calcification. The red arrow indicates the guidewire tip; yellow arrow, guidewire shaft; white line, calcification; and yellow area, plaque behind calcification.

An 8-F backup-type guiding catheter (Asahi Intecc) was engaged in the left coronary artery for the antegrade approach, and a 7-F Amplatz left-type guiding catheter (Medtronic) was engaged in the right coronary artery to visualize the distal true lumen. Tip injection from the right coronary artery demonstrated that the epicardial collateral channels were unsuitable for a retrograde approach because of their small diameter, tortuosity, and unfavorable origin; therefore, an antegrade strategy, including IVUS-guided wiring, was adopted as the final option (Figure 2A).

The LCX entry was stumpless, and IVUS demonstrated massive calcification occupying the CTO entry site (Figures 2B and 2C, Video 5). Initial attempts to puncture the calcified entry using Gaia Next 4 (GN4), Conquest Pro 12, and Conquest Pro 8-20 (all from Asahi Intecc), supported by Corsair Pro and Sasuke (Asahi Intecc), under IVUS-based 3D wiring guidance were unsuccessful because of impenetrable calcification (Figures 2D to 2F). Given that the calcified entry was too hard to penetrate with conventional guidewires and would likely hinder device delivery even if crossed, a “move the cap” strategy was adopted to avoid the calcified entry.

**Figure 3:**
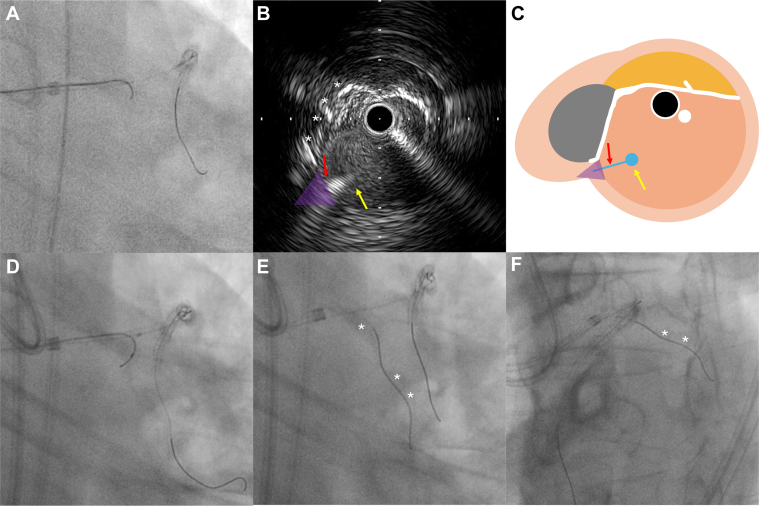
Angiographic and Intravascular Ultrasound Images When the “Move the Cap” Technique Was Performed (A) Gaia Next 4 successfully entered the extraplaque space under real-time intravascular ultrasound guidance. (B) Intravascular ultrasound image when Gaia Next 4 punctured the edge of the calcified area connecting the extraplaque space. The red arrow indicates the guidewire tip; yellow arrow, guidewire shaft; asterisk, calcification; and purple area, edge of calcification. (C) Intravascular ultrasound view. The red arrow indicates the guidewire tip; yellow arrow, guidewire shaft; white line, calcification; and purple area, edge of calcification. (D) Gladius EX traced the route that was created by Gaia Next 4. (E and F) Gladius EX following angiographic calcification. The asterisk indicates calcification.

Using IVUS-based 3D wiring, GN4 was intentionally directed toward the edge of the calcified area at the distal carina on the myocardial side of the LCX entry (Video 6). This allowed successful entry into the extraplaque space within the vessel architecture outside the calcified segment (Figures 3A to 3C).

After entry into the extraplaque space with GN4, the guidewire was exchanged for Gladius EX (Asahi Intecc) to follow the created tract and reduce the risk of perforation associated with GN4 (Figure 3D). Intentional extraplaque tracking with Gladius EX was then performed on the basis of angiographic calcification (Figures 3E and 3F).

**Figure 4:**
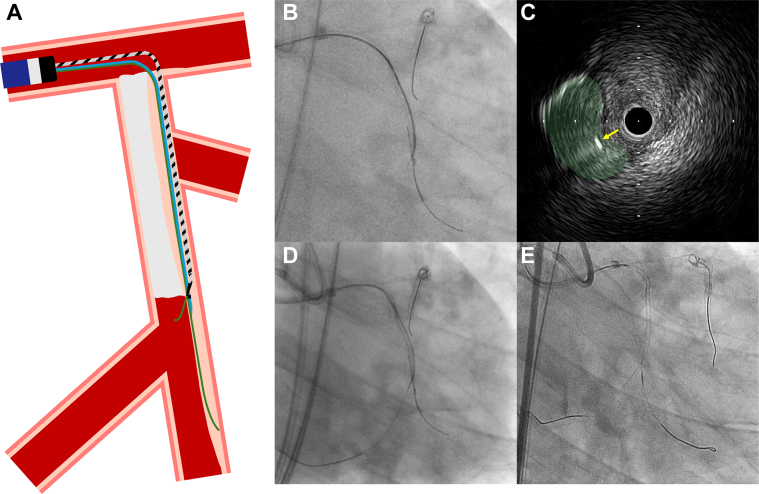
Angiographic and Intravascular Ultrasound Images Obtained During Tip Detection–Guided Antegrade Dissection Re-Entry (A) Illustration of tip detection–guided antegrade dissection re-entry. Corsair Pro and intravascular ultrasound catheters were placed in the extraplaque space to prevent massive calcification. Conquest Pro 12 Sharpened Tip successfully re-entered the distal true lumen using tip detection–guided antegrade dissection re-entry. (B) Conquest Pro 12 Sharpened Tip successfully re-entered the distal true lumen immediately before bifurcation using tip detection–guided antegrade dissection re-entry. (C) Intravascular ultrasound image when Conquest Pro 12 Sharpened Tip re-entered the distal true lumen. The green area indicates the distal true lumen; and yellow arrow, Conquest Pro 12 Sharpened Tip. (D) The workhorse wire easily passed the posterior branch of the left circumflex artery after tip detection–guided antegrade dissection reentry. (E) Another workhorse wire easily passed through the posterolateral branch of the left circumflex artery using a dual-lumen microcatheter.

The initial attempt of TD-ADR between the first and second CTO segments was unsuccessful because of severe calcification. Therefore, TD-ADR was performed at the distal end of the second CTO. Conquest Pro 12 Sharpened Tip (Asahi Intecc) successfully entered the true lumen just proximal to the distal bifurcation (Figures 4A to 4C), allowing the passage of a workhorse guidewire into the distal true lumen (Figures 4D and 4E, Video 7).

**Figure 5:**
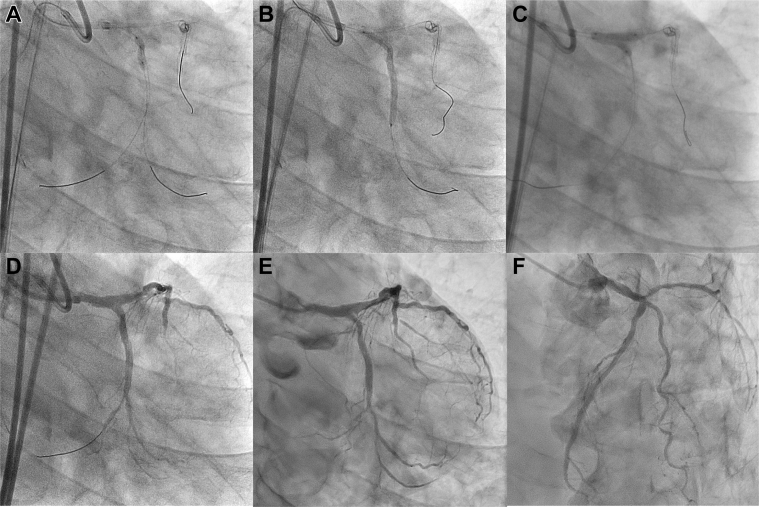
Angiographic Images After Guidewire Crossing and at 1-Year Follow-Up (A) Predilation using a 2.5-mm balloon. (B) A 2.5-mm sirolimus-eluting stent was implanted from the left main trunk to the left circumflex artery. (C) Kissing balloon inflation was performed after stent implantation. (D) Final angiography immediately after the procedure demonstrated satisfactory coronary flow. (E and F) Follow-up angiography at 1 year showed good lesion patency without restenosis.

After predilation with a 2.5-mm balloon (Figure 5A), a 2.5-mm sirolimus-eluting stent was implanted from the left main trunk to the LCX (Figure 5B). After recrossing into the LAD, kissing balloon inflation with 3.5-mm and 2.5-mm balloons was performed to optimize the LAD ostium (Figure 5C). Final angiography demonstrated adequate coronary flow without complications (Figure 5D, Video 8).

## Follow-Up

The patient remained asymptomatic after PCI, and follow-up angiography at 1 year demonstrated no evidence of restenosis (Figures 5E and 5F).

## Discussion

Severe calcification, particularly at the proximal cap, complicates CTO PCI by increasing the likelihood of guidewire and device crossing failure.^5^ When the proximal cap remains impenetrable despite guidewire escalation and adequate backup support, alternative strategies include a retrograde approach, the “move the cap” technique, or proximal cap modification using atherectomy or intravascular lithotripsy.^6^

The “move the cap” technique is an effective strategy for ambiguous or impenetrable proximal caps; however, a major limitation is the inability to control the entry site into the extraplaque space, which may increase the risk of compromising side branches. In contrast, IVUS-guided “move the cap” in this case enabled selection of an optimal puncture site on the basis of real-time IVUS imaging. Visualization of the guidewire tip allowed intentional entry into the extraplaque space at the desired location, potentially reducing the risk of perforation and unnecessary expansion of the extraplaque space.

Differentiating the extraplaque space from the intraplaque space on IVUS can sometimes be challenging. In the present case, the target was presumed to be located outside the calcified plaque but within the vessel architecture and was identifiable on IVUS, allowing precise puncture (Figures 3B and 3C). In flush-type proximal caps with an important side branch, puncturing close to the distal carina is essential to avoid side branch compromise. After entry with a high-penetration-efficacy guidewire, prompt exchange for a nontapered guidewire is advisable to facilitate blunt advancement and reduce the risk of perforation.

The “move the cap” technique is usually followed by re-entry strategies such as ADR, retrograde dissection and re-entry, subintimal tracking and re-entry,^7^ or limited antegrade subintimal tracking^8^ When a retrograde approach is high-risk or not feasible, ADR becomes essential. However, subintimal tracking and re-entry and limited antegrade subintimal tracking may cause distal extraplaque expansion, leading to longer stent lengths and possible side-branch loss. Although device-based ADR techniques are commonly used, their success rates are still limited, and controlling the re-entry side precisely can be challenging.

TD-ADR is increasingly recognized as an effective and reliable technique.^9^ TD-ADR allows real-time visualization of the guidewire tip with IVUS, facilitating re-entry at a specified location. In this case, because a side branch was located at the distal cap, TD-ADR enabled re-entry just proximal to the bifurcation, thus preserving the distal side branch.

When the “move the cap” technique is used on proximal bifurcation lesions, stenting across the bifurcation is often necessary. In this case, because no significant stenosis was observed at the LAD ostium, a single crossover stent from the left main trunk to the LCX was placed. More complex stenting strategies might be needed depending on lesion characteristics.

To our knowledge, this is the first reported case of successful CTO revascularization with an impenetrable proximal cap using an IVUS-guided “move the cap” technique based on the tip detection method combined with TD-ADR.

## Conclusions

The “move the cap” technique is an effective strategy for CTO lesions with an impenetrable proximal cap. IVUS-based 3D wiring can facilitate precise and safer entry into the extraplaque space, improving procedural success in complex CTO interventions.

## Funding Support and Author Disclosures

The authors have reported that they have no relationships relevant to the contents of this paper to disclose.

## Appendix

Video 1 Baseline Coronary Angiography of the Left Coronary Artery From the Left Anterior Oblique/Caudal View

Video 2 Baseline Coronary Angiography of the Left Coronary Artery From the Anteroposterior View

Video 3 Baseline Coronary Angiography of the Right Coronary Artery From the Right Anterior Oblique/Caudal View

Video 4 Baseline Coronary Angiography of the Right Coronary Artery From the Left Anterior Oblique/Cranial View

Video 5 Intravascular Ultrasound of the Chronic Total Occlusion Entry Site

Video 6 Intravascular Ultrasound–Guided “Move the Cap” Technique

Video 7 Intravascular Ultrasound–Guided Antegrade Dissection Re-Entry Using the Tip Detection Method

Video 8 Final Coronary Angiography
